# Comparison of endoscopic retrograde cholangiopancreatography with laparoscopic surgery for patients with mild and moderately severe acute biliary pancreatitis

**DOI:** 10.1016/j.heliyon.2024.e36216

**Published:** 2024-08-13

**Authors:** Chengsi Zhao, Zuoquan Wang, Yanrong Yao, Weijie Yao, Zuozheng Wang

**Affiliations:** a Department of Hepatobiliary Surgery, General Hospital of Ningxia Medical University, Yinchuan City, Ning Xia Province, China; b Department of General Surgery, the Third Affiliated Hospital of Xi'an Medical University, Xi'an City, Shan Xi Province, China

**Keywords:** Acute biliary pancreatitis, ERCP, Laparoscopic surgery, Treatment efficacy, Recurrence, Risk factors

## Abstract

Acute biliary pancreatitis (ABP) is an acute inflammatory reaction that occurs as a result of abnormal reflux of bile into the pancreatic duct, which activates pancreatic digestive enzymes to produce pancreatic auto-digestion.

**Objectives:**

To explore the advantages of Endoscopic Retrograde Cholangiopancreatography (ERCP) treatment compared with laparoscopic surgery in the management of patients with mild and moderately severe ABP, and to study the risk factors for recurrence of ABP and construct a risk prediction model to assist in resolving clinical decision-making and improving prognosis.

**Methods:**

Patients with mild and moderately severe ABP treated at General Hospital of Ningxia Medical University from January 1, 2019 to July 1, 2022 were reviewed. A total of 327 patients were enrolled according to the inclusion criteria and exclusion criteria. According to the different treatment modalities, they were divided into the group treated via ERCP (n = 239) and the group treated via laparoscopic surgery (n = 88). Statistical analyses were performed to compare the differences between the average levels of preoperative and postoperative blood routine and blood biochemical indexes, as well as the time of recovery from clinical symptoms, length of hospital stay, and postoperative complications between the two groups of patients. The 280 patients who participated in the follow-up were divided into the recurrence group (n = 130) and the non-recurrence group (n = 150) according to whether they had recurrence or not. Independent samples *t*-test and binary logistic regression were used to analyze the causative monofactors and risk factors of recurrent biliary pancreatitis, and then to construct the model and assess the predictive accuracy of the model.

**Results:**

On postoperative day 2, the incidence of local complications, Balthazar CT score, and the number of analgesia were lower in the patients in the group treated by ERCP than in the group treated by laparoscopic surgery (*P* < 0.001), and the duration of antibiotics, enzyme-suppressing medication, fasting, and hospital stay were shorter in the patients in the group treated by ERCP than in the group treated by laparoscopic surgery (*P* < 0.001). Personal history, gamma glutamyl transpeptidase (GGT), and treatment modality are risk factors for recurrence of biliary pancreatitis. The model constructed by combining GGT, personal history, and treatment modality had the best predictive ability for disease recurrence compared with the model with GGT, personal history, and treatment modality alone (area under the ROC curve 0.815).

**Conclusion:**

Compared with the laparoscopic surgery group, ERCP treatment can effectively relieve symptoms and restore gastrointestinal function in advance in patients with ABP, and reduce hospitalisation time and related complications. Personal history, GGT, and treatment modality are risk factors for recurrence of biliary pancreatitis. Patients can prevent recurrence by abstaining from smoking and alcohol, eating a healthy diet, and exercising appropriately.

## Highlights

• ERCP treatment can reduce hospitalisation time and related complications.
• Personal history, GGT, and treatment modality are risk factors for recurrence of biliary pancreatitis.

## 1 Introduction

Acute pancreatitis (AP) is one of the common acute abdominal conditions and its incidence is increasing worldwide due to the increased incidence of obesity and gallstones [1]. Acute biliary pancreatitis (ABP) is the most common type of acute pancreatitis [2]. Most patients with ABP have mild symptoms. However, about 20–25 % of patients may experience local or systemic complications and develop severe acute pancreatitis (SAP) with a mortality rate of about 2–10 % [3]. There are many causes of ABP, alcoholic ABP, hypertriglyceridemic ABP, and cholestatic ABP are more common in the clinic, and less commonly can be caused by surgical methods and α1-antitrypsin deficiency [[4], [5], [6], [7], [8]]. Ghalehnoei et al., investigated risk factors associated with post-ERCP pancreatitis (PEP) in two groups of patients and found that prophylactic pancreatic duct stenting and rectal indomethacin did not reduce possible PEP. prolonged deep intubation and pancreatic ductal dilatation of <10 mm could be considered as significant risk factors [9]. Therefore, it is particularly important to explore the risk factors affecting the recurrence of ABP, to guide the clinical assessment of the likelihood of recurrence and to develop individualised treatment and follow-up protocols.

There are conservative treatment and surgical treatment. The treatment principle of conservative treatment is to reduce the pancreatic and systemic inflammatory reaction and alleviate the clinical symptoms of patients, and clinically use the comprehensive treatment combining fasting and gastrointestinal decompression, fluid resuscitation, acid suppression, inhibition of pancreatic enzyme activity, antibiotics and anti-infections, relief of biliary spasm, and reasonable analgesia [10,11]. Surgical treatment, on the other hand, can be divided into (1) endoscopic surgical treatment, such as ERCP [12]; (2) laparoscopic surgical treatment, such as laparoscopic choledochotomy (LCBDE) + T-tube drainage [13], and laparoscopic cholecystectomy (LC), etc [14,15]; (3) open surgical treatment [16]; and (4) ultrasound-guided cholecystectomy with gallbladder puncture and drainage (PTGD), etc [17]. ERCP combined with abdominal ultrasound and ultrasound endoscopy can be used for the diagnosis of ABP [18]; in addition, combined with endoscopic sphincterotomy (EST), which can be used to incise the sphincter of Oddi to relieve pancreatic hypertension, contributes to a better prognosis of ABP [19,20]. Guidelines recommend urgent ERCP in patients with ABP [21]. The clinical efficacy of pancreatic duct stenting under ERCP for ABP has also been reported [22]. However, neither ERCP combined with EST nor performing pancreatic duct stenting has been widely recognised, and its advantages remain questionable [23]. In summary, we focus in this paper on the advantages of transendoscopic retrograde cholangiopancreatography (ERCP) treatment compared with laparoscopic surgery in the management of patients with mild and moderately severe acute biliary pancreatitis (ABP), as well as investigating the risk factors for recurrence of ABP, and constructing a risk prediction model to assist in solving the clinical decision-making and improving the prognosis.

## 2 Materials and methods

### 2.1 Research subjects

**Fig. 1:**
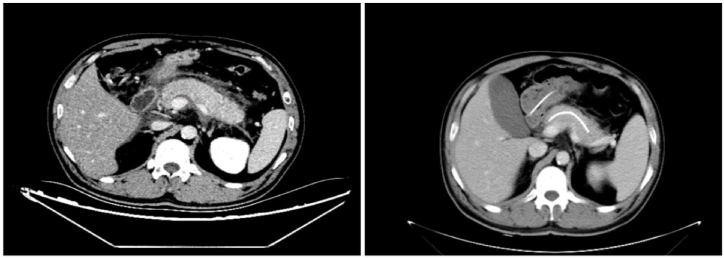
A 39-year-old male with moderate biliary pancreatitis was admitted to the hospital with marked thickening of the pancreas and accumulation of peripancreatic fluid. The peripancreatic fluid was completely absorbed after 5 days of pancreatic duct drainage.

**Fig. 2:**
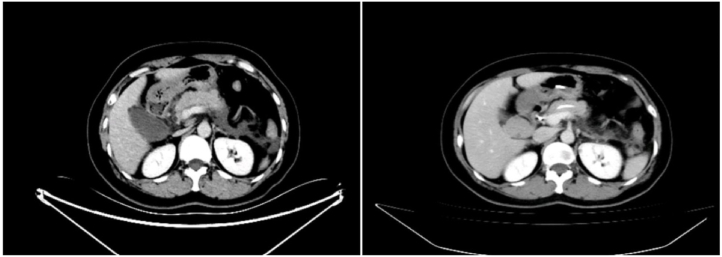
A 48-year-old woman with moderate biliary pancreatitis was admitted to the hospital with fluid accumulation around the pancreas, which decreased significantly after 5 days of pancreatic duct drainage.

Clinical data of ABP patients admitted to the General Hospital of Ningxia Medical University (China) between January 1, 2017 and July 1, 2022 were retrospectively analysed. Inclusion criteria: (1) diagnosis of ABP and first episode [24]; (2) surgery less than 72 h from admission; (3) moderate (Fig. 1) severe (Fig. 2) or Acute Physiology and Chronic Health (APACHE) II score of ≥8 according to the 2012 Revised Atlanta Classification of Pancreatitis [25]. Exclusion criteria: (1) pregnant and lactating women; (2) acute exacerbation of chronic pancreatitis; (3) poor general condition of the patients or inability to tolerate ERCP; (4) incomplete clinical data; (5) combination of chronic infections prior to admission; (6) combination of chronic organ failure; (7) immune deficiency; and (8) previous ERCP. Finally, 327 ABP patients were enrolled and divided into two groups according to the treatment method: the group treated by ERCP (n = 239) and the group treated by laparoscopic surgery (n = 88).

**Fig. 3:**
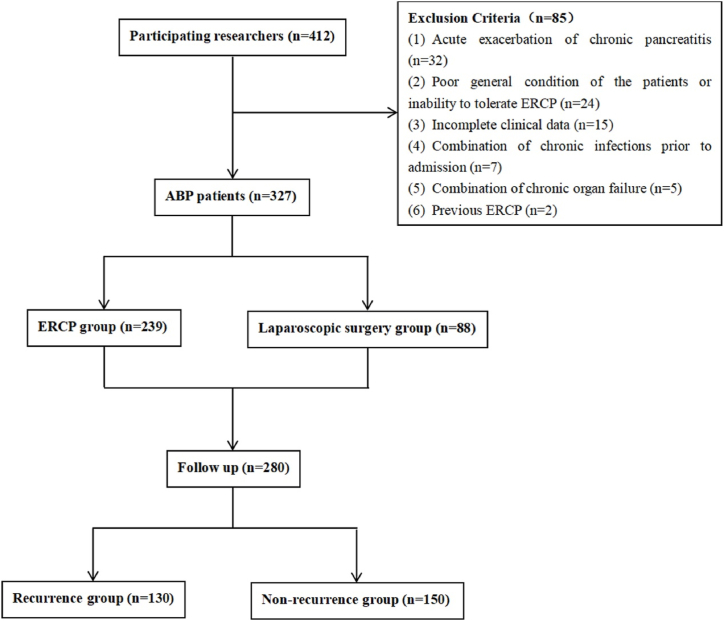
Flow chart.

280 patients who participated in the follow-up were divided into recurrence group (n = 130) and non-recurrence group (n = 150), according to whether they had relapsed or not (the follow-up time was ≥1 year from the initial onset of the disease, and the interval between the two relapses was at least 3 months) (Fig. 3).

The study was approved by the Hospital Ethics Committee of the General Hospital of Ningxia Medical University (Approval No. 2019-467), and patients were informed of the two treatment options in detail, and they all freely chose and signed the surgical and informed consent forms.

### 2.2 Sample size calculation

The diagnostic part of this study was tested by matching the ERCP group with the laparotomy treatment group, and the sample size estimation formula was as follows:$=\frac{z_{a}^{2}P\left(1-P\right)}{\delta^{2}}$.

Setting the expected index value: assuming that the significance level of the test α = 0.05, the error allowed for the experiment δ = 0.1, and p is the sensitivity or specificity of the diagnostic experiment to be evaluated, we applied sensitivity to estimate the sample size of the ERCP group, and specificity to estimate the sample size of the laparotomy treatment group, and set the sensitivity at 75 % and the specificity at 85 %. The minimum sample size required was calculated based on the expected index values set.

### 2.3 Treatment

All patients received the same standardised treatment upon admission and early treatment was based on the American Gastroenterological Association guidelines for the initial management of acute pancreatitis [26]. In addition to the above conservative treatment measures, according to the patient grouping the patients were treated with ERCP surgery in the trans-ERCP treatment group (n = 239) and laparoscopic surgery in the trans-laparoscopic surgery treatment group (n = 88). At the same time, if the patients were combined with acute cholangitis or biliary obstruction, ERCP was performed within 24 h after admission, and patients with suspected choledochal stones without jaundice underwent ERCP after MRCP to clarify the diagnosis.

### 2.4 Key and secondary indicators detection indicators in ERCP and laparoscopic surgery group

Key indicators: mainly including new systemic complications, occurrence of local complications, number of days of fasting, number of days of hospitalisation, duration of enzyme-suppressing drugs, number of analgesic sessions, duration of antibiotic use, and relapse.

Secondary indexes: white blood cells, amylase before and after treatment (48 h after surgery) were compared between the two groups, total bilirubin (TBIL), creatinine, transaminases, APACHE II [27] and Balthazar CT score [28].

### 2.5 Indicators for monitoring the recurrence group and the non-recurrence group

General clinical information: gender, age, body mass index (BMI), personal history (history of smoking, alcohol consumption, diabetes, fatty liver, cholecystitis), stones and obstruction, treatment modality, disease classification, severity score, and outcome.

Laboratory indices: TBIL, alanine aminotransferase (ALT), alanine transaminase (AST), alkaline phosphatase (ALP), gamma glutamyl transpeptidase (GGT), lactate dehydrogenase (LDH), and mean platelet volume (MPV).

### 2.6 Statistical analyses

SPSS 21.0 software was used for statistical analysis. Normally distributed measures were expressed as ($\bar{x}\pm s$), and comparisons between groups were made using the independent samples *t*-test. Non-normally distributed measurement information was expressed as Md (P25, P75) and comparisons between groups were made using the rank sum test. P25 and P75 correspond to the 75th and 25th percentile. Count data were expressed as frequency (%), and the χ^2^ test was used for comparison between groups. The analysis of risk factors was processed by binary Logistic regression analysis and the difference was considered statistically significant at *P* < 0.05.

## 3 Results

### 3.1 Basic characteristics of patients in the ERCP and translaparoscopic surgery groups

There were no significant differences in age distribution, Sex, BMI, Time from admission to surgery, AMYL, WBC, APACHE II and so on. The translaparoscopic surgery treatment group was 62.00 ± 13.15 years, the trans-ERCP surgical treatment group was 61.29 ± 14.93 years; the BMI in the translaparoscopic surgery treatment group was 24.27 ± 3.83, in the trans-ERCP surgical treatment group was 24.40 ± 3.81; Time from admission to surgery in the translaparoscopic surgery treatment group was 34 (24–48) h, in the trans-ERCP surgical treatment group was 30 (24–48) h, the difference between the two groups was not significant (P > 0.05) and the baseline levels were consistent and comparable (Table 1).

**Table 1:** Comparison of clinical data at the time of admission between patients in the group treated by ERCP and the group treated by laparoscopic surgery.

Variant	Translaparoscopic surgery treatment group (n = 88)	Trans-ERCP surgical treatment group (n = 239)
Age [years, (‾X ± S)]	62.00 ± 13.15	61.29 ± 14.93
Z	−0.418	
P	0.677	
Sex (n, %)		
Man	51 (57.95)	124 (51.88)
Woman	37 (42.05)	115 (48.12)
Z	0.953	
P	0.329	
BMI (‾X ± S)	24.27 ± 3.83	24.40 ± 3.81
Z	0.264	
P	0.792	
Balthazar CT [points, (‾X ± S)]	5.48 ± 1.50	5.13 ± 1.99
Z	−1.673	
P	0.096	
Combined organ failure (n, %)	25 (28.41)	79 (33.05)
Z	0.640	
P	0.424	
Variant	Translaparoscopic surgery treatment group (n = 88)	Trans-ERCP surgical treatment group (n = 239)
Time from admission to surgery [h, M (Q1-Q3)]	34 (24–48)	30 (24–48)
χ^2^	−1.412	
P	0.158	
AMYL [U/L, M (Q1-Q3)]	865.75 (550.35–1316.85)	913.50 (444.90–1349.00)
χ^2^	−0.322	
P	0.748	
WBC [10^9^/L, M (Q1-Q3)]	10.97 (7.99–15.13)	11.22 (8.11–14.89)
χ^2^	−0.138	
P	0.890	
APACHE II [points, M (Q1-Q3)]	10.00 (7.25–13.00)	10.00 (7.00–12.00)
χ^2^	−0.175	
P	0.861	
Creatinine [μmol/L,M(Q1-Q3)]	68.00 (52.85–80.48)	65.80 (55.95–80.40)
χ^2^	−0.174	
P	0.862	
TBIL [μmol/L,M(Q1-Q3)]	86.10 (50.15–117.85)	68.70 (41.55–114.30)
χ^2^	−1.567	
P	0.117	
ALT [U/L, M (Q1-Q3)]	257.10 (150.65–426.50)	243.80 (118.50–423.90)
χ^2^	−0.813	
P	0.416	
AST [U/L, M (Q1-Q3)]	160.87 (145.73–176.54)	158.25 (145.93–177.02)
χ^2^	−0.281	
P	0.612	

Note: AMYL, serum amylase; WBC, white blood cell count; TBIL, total bilirubin; ALT, alanine aminotransferase; AST, aspartate aminotransferase.

### 3.2 Comparison of the treatment effects of patients in the group treated by ERCP and the group treated by laparoscopic surgery at 2 days postoperatively

The test indexes such as AMYL, TBIL, AST, ALT, WBC, etc. were reviewed on the 2nd day after the operation in both groups, and there was a significant decrease in comparison with the preoperative period, and there was a statistically significant difference in the comparison of the mean levels of the indexes before and after the treatment of the two groups (*P* < 0.05) (Table 2), and the treatments were considered to be effective.

**Table 2:** Changes in general laboratory indices 2 days after surgery in the group treated by ERCP and the group treated by laparoscopic surgery.

Variant		Translaparoscopic surgery treatment group (n = 88)	Trans-ERCP surgical treatment group (n = 239)	χ^2^	P
AMYL [U/L, M (Q1-Q3)]	pre-treatment	865.75 (550.35–1316.85)	913.50 (444.90–1349.00)	−0.322	0.748
	post-treatment	403.24 (239.47–562.23)	478.57 (287.12–670.02)	−0.457	0.712
χ^2^		6.713	6.957		
P		0.012	0.010		
WBC [10^9^/L, M (Q1-Q3)]	pre-treatment	10.97 (7.99–15.13)	11.22 (8.11–14.89)	−0.138	0.890
	post-treatment	9.83 (6.46–13.20)	9.17 (6.24–12.10)	−0.159	0.824
χ^2^		5.758	5.913		
P		0.019	0.018		
TBIL [μmol/L, M (Q1-Q3)]	pre-treatment	86.10 (50.15–117.85)	68.70 (41.55–114.30)	−1.567	0.117
	post-treatment	54.24 (26.47–82.01)	49.70 (36.29–63.11)	−1.258	0.206
χ^2^		8.293	7.849		
P		0.006	0.007		
ALT [U/L, M (Q1-Q3)]	pre-treatment	257.10 (150.65–426.50)	243.80 (118.50–423.90)	−0.813	0.416
	post-treatment	168.27 (72.52–264.02)	141.41 (66.36–216.46)	−0.828	0.425
χ^2^		6.748	7.112		
P		0.011	0.0010		
AST [U/L, M (Q1-Q3)]	pre-treatment	160.87 (145.73–176.54)	158.25 (145.93–177.02)	−0.281	0.612
	post-treatment	102.28 (69.14–135.42)	95.36 (62.48–128.54)	−0.297	0.629
χ^2^		5.952	6.258		
P		0.018	0.0016		

Note: AMYL, blood amylase; WBC, white blood cell count; TBIL, total bilirubin; ALT, alanine aminotransferase; AST, aminotransferase.

On postoperative day 2, patients in the trans-ERCP-treated group had a lower incidence of local complications, Balthazar CT score, and number of analgesia than those in the trans-laparoscopic surgery-treated group (5.44 % vs. 12.50 %, χ^2^ = 4.715, *P* = 0.030), (3.79 ± 1.46 points vs. 4.26 ± 1.12 points, *t* = −3.092, *P* = 0.002), [1 (1–2) vs 3 (2–3), *Z* = −11.213, *P* < 0.001] (Table 3), and the duration of antibiotic use, enzyme-suppressing drugs, fasting time, and hospital stay of patients in the group treated by ERCP were shorter than that of the patients in the group treated by laparoscopic surgery: [5 (4–7) vs 7 (5–9), *Z* = −4.688, *P* < 0.001], [2 (1–3) vs 6 (5–7), *Z* = −10.344, *P* < 0.001], [4 (3–6) vs 6 (5–8), *Z* = −5.094, *P* < 0.001], [7 (6–10) vs 9 (7–11), *Z* = −3.639, *P* < 0.001] (Table 3).

**Table 3:** Comparison of post-treatment clinical data of patients in the group treated via ERCP and the group treated via laparoscopic surgery.

Variant	Translaparoscopic surgery treatment group (n = 88)	Trans-ERCP surgical treatment group (n = 239)
New systemic complications (n, %)	11 (12.50)	20 (8.37)
Z	1.280	
P	0.258	
Local complications (n, %)	11 (12.50)	13 (5.44)
Z	4.715	
P	0.030	
Balthazar CT [Points, (‾X ± S)]	4.26 ± 1.12	3.79 ± 1.46
*t*	−3.092	
P	0.002	
Duration of antibiotic use [d, M (Q1-Q3)]	7 (5–9)	5 (4–7)
χ^2^	−4.688	
P	＜0.001	
Number of analgesia [times, M (Q1-Q3)]	3 (2–3)	1 (1–2)
χ^2^	−11.213	
P	＜0.001	
Duration of use of enzyme-suppressing drugs [d, M (Q1-Q3)]	6 (5–7)	2 (1–3)
χ^2^	−10.344	
P	＜0.001	
Fasting time [d, M (Q1-Q3)]	6 (5–8)	4 (3–6)
χ^2^	−5.094	
P	＜0.001	
Length of hospitalisation [d, M (Q1-Q3)]	9 (7–11)	7 (6–10)
χ^2^	−3.639	
P	＜0.001	
Hospitalisation costs [yuan, M (Q1-Q3)]	34877 (27501–41977)	35435 (30941–42538)
χ^2^	−1.011	
P	0.312	

### 3.3 Baseline comparison of relapse and non-relapse groups

A total of 280 patients were included in the study, 70 males and 60 females in the relapse group (n = 130), aged 24–90 years (mean 60.95 ± 16.81 years), with a BMI of 17.16–43.08 kg/m^2^ (mean 24.21 ± 3.67 kg/m^2^) (Table 4); and 80 males and 70 females in the non-relapse group (n = 150), aged 25–88 years (mean 61.12 ± 17.93 years), with a BMI of 16.89–42.26 kg/m^2^ (mean 24.45 ± 3.71 kg/m^2^) (Table 4). There was no statistically significant difference between the two groups in terms of gender, age, and BMI (p-value all >0.05) (Table 4).

**Table 4:** Baseline comparison of patients in the relapse and non-relapse groups.

Variant	Recurrent group (n = 130)	Non-recurrent group (n = 150)	t	P
Age [years, (‾X ± S) ]	60.95 ± 16.81	61.12 ± 17.93	−0.456	0.668
Sex (n, %)			0.814	0.415
Man	70（53.85）	80（53.33）		
Woman	60（46.15）	70（46.67）		
BMI (‾X ± S)	24.21 ± 3.67	24.45 ± 3.71	1.835	0.072

Note: BMI, body mass index.

### 3.4 Univariate comparison of case data between the recurrence group and the non-recurrence group

The prevalence of fatty liver, GGT values, biliary obstruction rate, presence of juxtapapillary diverticulum percentage, gallbladder neck stone rate, and presence of history of alcohol consumption and smoking were higher in the recurrent group than in the non-recurrent group; the stone-free rate and EST treatment rate were higher in the non-recurrent group than in the recurrent group, and the differences were statistically significant (*P* < 0.05) (Table 5). The differences between the two groups in terms of history of diabetes, TBIL, ALT, AST, ALP, MPV, cholestasis rate, prevalence of cholecystitis, rate of bile mud-like stones, length of hospitalisation, ranson score, BISAP score, and MCTSI score were not statistically significant (*P* > 0.05) (Table 5).

**Table 5:** Univariate analysis of general data of patients in the relapse and non-relapse groups.

		Recurrent group (n = 130)	Non-recurrent group (n = 150)
Laboratory indicators			
TBIL (μmol/L)		32.712	28.693
Z		0.578	
P		0.572	
ALT (U/L)		137.89	152.05
Z		1.571	
P		0.121	
AST (U/L)		136.96	158.68
Z		1.248	
P		0.225	
ALP (U/L)		126.94	127.37
Z		0.721	
P		0.482	
GGT (U/L)		284.97	248.61
Z		2.375	
P		0.019	
MVP (fl)		11.415	11.147
Z		0.142	
P		0.893	
Stone site (n, %)	Non	5 (3.85)	15 (10.00)
	Gall bladder	60 (46.15)	80 (53.33)
	Neck of the gallbladder	34 (26.15)	8 (5.33)
	Common bile duct	20 (15.38)	32 (21.33)
	Gallbladder & Gallbladder Neck	4 (3.08)	3 (2.00)
	Gallbladder & Duct	7 (5.38)	12 (8.00)
χ^2^		13.0191	
P		0.016	
Personal history (n, %)	Smoking	20 (15.38)	14 (9.33)
	Drinking	4 (3.08)	15 (10.00)
	Smoking & Drinking	48 (36.92)	27 (18.00)
	Non	62 (47.69)	94 (62.67)
χ^2^		8.512	
P		0.034	
Diabetes (n, %)		13 (10.00)	23 (15.33)
χ^2^		0.691	
P		0.405	
Fatty liver (n,%)		41 (31.54)	20 (13.33)
χ^2^		5.815	
P		0.015	
Cholecystitis (n, %)		80 (61.54)	80 (53.33)
χ^2^		0.856	
P		0.358	
Cholestasis (n, %)		21 (16.15)	30 (20.00)
χ^2^		0.228	
P		0.647	
Slush-like stone (n, %)		28 (21.54)	50 (33.33)
χ^2^		2.066	
P		0.159	
Periportal diverticulum (n, %)		24 (18.46)	10 (6.67)
χ^2^		4.627	
P		0.036	
Biliary tract obstruction (n, %)		13 (10.00)	0 (0.00)
χ^2^		4.397	
P		0.039	
Number of stones		2.98	3.01
χ^2^		−0.702	
P		0.486	
Length of hospitalisation		9.64	9.12
χ^2^		−0.835	
P		0.407	
Severity		1.49	1.00
χ^2^		−2.332	
P		0.021	
Ranson (points)		1.47	1.39
χ^2^		−0.355	
P		0.725	
BISAP (points)		1.00	1.00
χ^2^		−0.237	
P		0.821	
MCTSI (points)		2.99	2.00
χ^2^		−1.559	
P		0.124	
Treatment modality (n, %)	Conservative treatment	106 (81.54)	24 (18.46)
	EST	92 (61.33)	58 (38.67)
χ^2^		4.678	
P		0.033	

Note: Number of stones: 1 = absent, 2 = single, 3 = multiple, 4 = cholestatic; severity: 1 = mild, 2 = moderate, 3 = moderately severe, 4 = severe.TBIL, total bilirubin; ALT, alanine aminotransferase; AST, azelaic transaminase; ALP, alkaline phosphatase; GGT, gamma glutamyltranspeptidase; MPV, mean platelet volume; EST, Endoscopic sphincterotomy.

### 3.5 Analysis of risk factors affecting relapse

Binary logistic regression was used to analyze the variables of GGT, presence of fatty liver, location of stones, presence of biliary obstruction, presence of juxtapetal diverticulum, treatment modality, severity, and personal history, and the results showed that personal history, GGT, and treatment modality were the independent risk factors for the recurrence of ABP (Table 6). The greater the value of GGT, the greater the likelihood of recurrence; patients who had a history of smoking and drinking had a greater likelihood of recurrence than those who did not; patients who did not receive EST surgery had a higher risk of recurrence than those who opted for EST surgery (Table 6). Patients with a history of both smoking and alcohol consumption were more likely to relapse than those without a history of smoking and alcohol consumption, and patients not treated with EST surgery had a higher risk of relapse than those who opted for EST surgery (Table 6).

**Table 6:** Multifactorial logistic regression analysis of ABP risk factors.

	Variant	Regression coefficient	P	OR
GGT	0.003	0.015	1.003	1.000–1.005
Treatment	1.527	0.013	4.596	1.387–15.182
Personal history	1.213	0.010	3.356	1.351–8.342
Constant	−2.357	0.001	0.098	–

The model was constructed as In (.) = -2.356 + 0.003*GGT value + 1.527*Treatment modality (EST = 1, Conservative treatment = 0)+1.213*Personal history (1 = Smoking, 2 = Drinking, 3 = Smoking and Drinking, 4 = Neither Smoking nor Drinking.

Note: For the reference category, select "vs. last". " comparison. gGT, gamma glutamyl transpeptidase.

### 3.6 Comparison of model predictive capabilities

Considering confounding effects and interactions between factors, the predictive ability of the model constructed by combining GGT, personal history, and treatment modality to predict disease recurrence was compared with the predictive ability of GGT, personal history, and treatment modality alone, and it was found that the area under the ROC curve of the constructed model was 0.815, (95 % CI 0.672–0.848), and Homer-Lemeshow goodness-of-fit test: P = 0.069 (>0.05) (Table 7). The area under the ROC curve for GGT, personal history, and treatment modality alone to predict disease recurrence were 0.638 (95 % CI 0.532–0.746), 0.421 (95 % CI 0.327–0.531), and 0.434 (95 % CI 0.337–0.541), respectively (Table 7). It was suggested that the three indicators of combined GGT, personal history, and treatment modality had the best predictive ability of whether the disease recurred, with a sensitivity of 69.5 %, a specificity of 78.7 %, and a Yoden index of 0.489 (Table 7). In addition, the optimal threshold value of GGT for predicting disease recurrence was calculated as 391.2 U/L based on the Jordon's index, which means that when the GGT model alone is used to predict whether the disease is recurrent or not, the accuracy of predicting the patient's disease recurrence is the highest when the GGT is greater than 391.2 U/L.

**Table 7:** Area under the curve for GGT, personal history, treatment modality and predictive probability.

Test Outcome Variables	Area	Standard error a	Gradual progress Sig.b	95 % CI	Specificity	Sensitivity	Yoden index
GGT	0.638	0.051	0.018	0.638 (0.532, 0.746)	69.5 %	73.4 %	0.413
Personal history	0.421	0.052	0.097	0.421 (0.327, 0.531)	65.2 %	78.7 %	0.428
Treatment	0.434	0.052	0.156	0.434 (0.337, 0.541)	61.9 %	68.2 %	0.489
Predictive probability	0.815	0.045	0.000	0.815 (0.672, 0.848)	43.7 %	57.5 %	0.396

Note: GGT, gamma glutamyl transpeptidase.

## 4 Discussion

In this research, 88 patients with ABP who underwent laparoscopic surgery were admitted to the hospital for conservative treatment to control their condition and then underwent surgery at an early stage, and there was a significant decrease in blood routine and blood biochemistry indexes after treatment compared with those before surgery (*P* < 0.05), and the treatment was effective. There is a great deal of controversy regarding the timing of laparoscopic surgery. Early opinions pointed out that for patients with mild ABP, after their conditions are controlled and reduced by conservative treatment, postponing surgical treatment, the surgical risk, surgical difficulty and the incidence of postoperative complications can be reduced [[29], [30], [31]], consistent with this research. However, in another study, cholecystectomy within 48 h significantly reduced the length of hospital stay in patients with ABP, and there was no significant difference in the conversion rate, operative time and complication rate [32].

Open and laparoscopic surgical treatment still has the disadvantages of long operation time, higher anaesthesia and surgical risk to be endured as well as a greater blow to the patient's organism, whereas endoscopic surgery, ERCP, has the advantages of balancing examination and treatment, less trauma [33]. Studies by experts and scholars have shown that the degree of necrosis of pancreatic tissue in patients with ABP treated with ERCP is effectively controlled, the length of hospital stay is greatly reduced, the number of deaths due to ABP has also been reduced [34,35]. In this research, 239 patients with ABP who underwent ERCP treatment were treated within 72 h of the onset of the disease, there was a significant decrease in blood routine and blood biochemistry indexes after treatment compared with the preoperative period (P < 0.05), the treatment was effective, the results of this study are consistent with the previous ones.

It has been concluded that ERCP treatment of ABP, compared with laparoscopic surgery, has a greater advantage in improving the recovery of patients' clinical symptoms and related blood sampling indexes, the complication rate is lower, which has a significant therapeutic effect [36]. Hormati et al. [37], found that the addition of UDCA to CBD stenting resulted in a reduction in stone size, which in turn facilitated stone expulsion and could be used as a first-line treatment for patients with large and multiple CBD stones. The following year, they conducted a randomised controlled clinical trial in 40 patients with biliary pancreatitis and found that common bile duct (CBD) stenting in patients with biliary pancreatitis combined with gallstones reduced the risk of gallstone recurrence and remobilisation in cases of delayed cholecystectomy [38]. This is consistent with the results obtained in this study, on the 2nd postoperative day, the incidence of local complications, Balthazar CT score, the number of analgesia in the patients in the group treated by ERCP were lower than those in the group treated by laparoscopic surgery (P < 0.001), the duration of antibiotics, enzyme-suppressing medications, fasting time and hospital stay in the patients in the group treated by ERCP were shorter than that in the patients in the group treated by laparoscopic surgery shortened (*P* < 0.001).

Recurrent acute biliary pancreatitis (RABP) is a recurrent episode of ABP in the clinic after evidence of chronic pancreatitis has been excluded, accounting for approximately 20–30 % of all ABP patients [39]. In this study, we collected clinical data from 280 patients, all of whom had their first episode of ABP, followed them up by telephone and SMS, then divided them into recurrent and non-recurrent groups according to the presence or absence of disease recurrence. The aim was to discover the risk factors affecting the recurrence of ABP and to construct a clinical risk prediction model, thus facilitating the accurate prediction of the recurrence rate of the disease at the first episode of pancreatitis, formulating an individualised follow-up system, proposing correct life guidance, assisting in resolving clinical decisions and improving the prognosis. A retrospective analysis of all the variables collected by independent samples *t*-test revealed that the prevalence of fatty liver, GGT, biliary obstruction, presence of juxtapical diverticulum, gallbladder neck stones, the number of people with a history of smoking and alcohol consumption were higher in the recurrence group than in the control group. The stone-free rate and EST surgical treatment rate were higher in the non-recurrent group than in the recurrent group, the difference was statistically significant (*P* < 0.05). Multifactorial logistic regression analysis of the above statistically different variables revealed that personal history, GGT and treatment modality were risk factors for RABP. At the same time, patients with a history of smoking and drinking had a higher risk of RABP than those with no bad habits, suggesting that smoking and drinking are significant risk factors for the recurrence of ABP, quitting smoking and drinking is conducive to reducing the chances of ABP recurrence. The risk of recurrence of ABP was higher in patients treated conservatively than in patients treated with EST surgery, therefore, EST surgery is an effective treatment to reduce biliary pancreatitis. This result is consistent with the findings of Ki Bae Bang et al. [40].

Our study also has many shortcomings, the sample size of the study is small, not a large RCT, as prospective study, and there is a certain population bias, which leads to a certain degree of chance in the data collected, and it is not broadly representative. In future studies, more treatment modalities could be included and subgroups of patients with pancreatitis could be set up to compare the differences in prognosis and incidence of RABP between different treatment modalities for different degrees of severity. Secondly, as the period considered in this study partially overlaps with the pandemic, there have been significant changes in the provision of healthcare and medical services as well as in the course of certain diseases during this period. Although we have endeavoured to remove as much interference as possible, the clinical data obtained in this study have some limitations. Therefore, in the next study, we hope to be able to include a large number of samples through a multicentre collaboration to re-engage clinical data collection and risk model prediction.

In conclusion, compared with the laparoscopic treatment group, ERCP treatment can effectively relieve symptoms and restore gastrointestinal function in advance in patients with ABP, reduce hospitalisation time and related complications. Personal history, GGT and treatment modality are risk factors for recurrence of biliary pancreatitis. Patients can prevent recurrence by abstaining from smoking and alcohol, eating a healthy diet, and exercising appropriately.

## Funding

This research was supported by the Key Scientific Research Project of the Health and Family Planning Commission of the Autonomous Region (2023-NWKYT-021), China; the Central Guiding Local Science and 10.13039/100006180Technology Development Fund Project (2023FRD05009), China; the Autonomous Region's high-level Science and Technology Innovation Leading Talents (2021GKLRLX04), China; and the Ningxia Autonomous Region's Science and Technology Benefit Program (2021CMG03013), China.

## Data availability statement

The datasets generated during and/or analysed during the current study are available from the corresponding author on reasonable request.

## Ethics approval and consent to participate

The study was approved by the Hospital Ethics Committee of the General Hospital of Ningxia Medical University (Approval No. 2019-467), and patients were informed of the two treatment options in detail, and they all freely chose and signed the surgical and informed consent forms.

## Consent for publication

Not applicable.

## CRediT authorship contribution statement

**Chengsi Zhao:** Writing – original draft. **Zuoquan Wang:** Writing – original draft. **Yanrong Yao:** Formal analysis. **Weijie Yao:** Formal analysis. **Zuozheng Wang:** Conceptualization.

## Declaration of competing interest

The authors declare that they have no known competing financial interests or personal relationships that could have appeared to influence the work reported in this paper.
